# Cardiovascular Risks of COVID-19 Therapeutics: Integrated Analysis of FAERS, Electronic Health Records, and Transcriptomics

**DOI:** 10.3390/ph19040574

**Published:** 2026-04-02

**Authors:** Xinran Zhu, Suguna Aishwarya Kuppa, Gibret Umeukeje, Robert Morris, Lan Bui, Kun Bu, Jie Zhang, Jin Wei, Feng Cheng

**Affiliations:** 1 Department of Pharmaceutical Sciences, College of Pharmacy, University of South Florida, Tampa, FL 33612, USA; xinranzhu@usf.edu (X.Z.); skuppa@usf.edu (S.A.K.); gibreti@gmail.com (G.U.); rpmj528@gmail.com (R.M.); 2 Department of Mathematics & Statistics, College of Art and Science, University of South Florida, Tampa, FL 33620, USA; buil@usf.edu (L.B.); kunbu@usf.edu (K.B.); 3 Section of Nephrology, Department of Medicine, Boston University Chobanian & Avedisian School of Medicine, Boston, MA 02118, USA; jzhang7@bu.edu (J.Z.); jwei7@bu.edu (J.W.)

**Keywords:** FAERS, pharmacovigilance, TriNetX, EHR, COVID-19 drugs, remdesivir, Paxlovid, REGEN-COV, bradycardia, ADE

## Abstract

**Background/Objectives**: The purpose of this study was to investigate the association between cardiovascular adverse drug events (ADEs) and the use of COVID-19 medicines. **Methods**: The analyses were conducted by leveraging pharmacovigilance data from the Food and Drug Authority (FDA) Adverse Event Reporting System (FAERS) and TriNetX electronic health records (EHRs). Transcriptomic data from human embryonic stem cell-derived cardiomyocytes (hESC-CMs) exposed to remdesivir were analyzed to provide supportive biological context for the observed cardiovascular safety signals. **Results**: Comparative analysis of three approved COVID-19 therapies revealed that COVID-19 patients treated with remdesivir had a higher risk of cardiovascular events than those treated with Paxlovid or REGEN-COV. FAERS analysis further indicated that bradycardia, hypotension, and cardiac arrest were the most frequently reported cardiovascular events associated with remdesivir, which was validated by propensity score-matched EHR data. These findings suggest an association between remdesivir exposure and increased cardiovascular ADEs relative to other COVID-19 therapies. Sex-stratified analysis using FAERS and EHR did not show strong sex-dependent patterns for remdesivir-associated cardiovascular ADEs. Age-stratified analyses of EHR data showed age-associated variation across the three cardiovascular ADEs. Bradycardia displayed a non-uniform pattern with higher prevalence in the youngest and oldest age groups, hypotension showed an overall age-associated increase, and cardiac arrest showed only a weak age-associated effect. Pathway enrichment analysis on transcriptomic data revealed that the “cGMP-PKG signaling pathway”, “dilated cardiomyopathy”, and “calcium signaling pathway” were enriched among genes up-regulated by remdesivir exposure. **Conclusions**: In summary, our integrated analysis of pharmacovigilance, EHR, and transcriptomic data provides convergent evidence for associations between remdesivir and cardiovascular ADEs and offers biological context into these associations.

## 1. Introduction

Since 2019, COVID-19 continues to generate substantial clinical burden, particularly among older adults and patients with cardiovascular disease, chronic lung disease, chronic kidney disease, cerebrovascular disease, and other risk factors for progression [1]. While vaccination has reduced severe outcomes, effective antivirals and antibody-based therapies remain central to reduce hospitalization and death in high-risk patients. As COVID-19 has been reported to cause cardiovascular complications including myocardial injury, arrhythmia, thromboembolic events, and heart failure [2], which elevates cardiovascular ADEs associated with COVID-19 therapies, it is clinically important to systemically evaluate the cardiovascular safety profiles of COVID-19 therapies. This study focused on three widely used and mechanistically distinct COVID-19 drugs: remdesivir, Paxlovid, and REGEN-COV, each of which reached large-scale clinical use.

Remdesivir, sold under the brand name Veklury and produced by Gilead Science, is a prodrug that is subsequently metabolized into an adenosine triphosphate analog with broad-spectrum activity against a number of RNA viruses including Severe Acute Respiratory Coronavirus-2 (SARS-CoV-2) [3]. Remdesivir was issued Emergency Use Authorization (EUA) by the FDA on 1 May 2020, for patients presenting with severe COVID-19 infection [4]. Due to the surge of the highly infectious Omicron variant as well as the decreased efficacy of monoclonal antibody treatment, the FDA expanded the approved usage of remdesivir in January 2022 for non-hospitalized patients that are at high risk of progressing to severe COVID-19, which may result in hospitalization or death [5]. In addition, remdesivir has practical value in clinical settings because it can be used alongside therapies that target complementary mechanisms, including glucocorticoids (e.g., dexamethasone), immunomodulators (e.g., baricitinib and tocilizumab), and other antiviral or investigational combination strategies [6,7,8]. The active triphosphate metabolite of remdesivir targets the RNA-dependent RNA polymerase (RdRp) of RNA viruses including SARS-CoV-2 [9]. The active triphosphate of remdesivir, termed the remdesivir triphosphate, is an analog of adenosine triphosphate. Incorporation of the remdesivir monophosphate into a growing RNA chain results in delayed chain termination in which transcription is stalled after the incorporation of three additional nucleotides, ultimately inhibiting viral replication [10]. Despite the conflicting reports on the efficacy of remdesivir in COVID-19 patients, the drug is still widely administered as a COVID-19 treatment with approximately $5.6 billion in sales reported by Gilead in 2021 and approximately $1.5 billion reported in the Q1 sales report [11]. Besides intravenous remdesivir, orally available remdesivir-related antivirals are also under active investigation. For example, obeldesivir (GS-5245), an oral prodrug related to the same antiviral nucleoside scaffold, has been developed to improve feasibility, and additional formulation strategies, including lipid-based carriers and nanoparticle delivery systems, have also been explored to enhance oral bioavailability [12,13,14].

Nirmatrelvir/ritonavir, with the brand name Paxlovid, is an oral antiviral combination in which nirmatrelvir is the active SARS-CoV-2 inhibitor and ritonavir is a pharmacokinetics enhancer via CYP3A inhibition to increase systemic exposure of nirmatrelvir [15]. Paxlovid was issued Emergency Use Authorization (EUA) by the FDA on 22 December 2021, for the treatment of mild-to-moderate COVID-19 in patients at high risk of progression to severe disease. Subsequently, full FDA approval was granted on 25 May 2023. Mechanistically, nirmatrelvir is a reversible covalent peptidomimetic inhibitor of the SARS-CoV-2 main protease (M^pro^), thereby blocking the proteolytic processing of viral polyproteins that is essential for replication [16]. Clinical efficacy was established in the randomized trial, in which early treatment with nirmatrelvir plus ritonavir substantially reduced the risk of COVID-19-related hospitalization or death among high-risk outpatients without evident safety concerns. Paxlovid has remained a widely utilized antiviral drug, with 2,103,570 unique prescriptions ordered from 22 December 2021 to 12 August 2023 in an analysis [17].

Casirivimab/imdevimab, sold under the brand name REGEN-COV and developed by Regeneron Pharmaceuticals, is a neutralizing monoclonal antibody (mAb) cocktail consisting of two recombinant human IgG1 antibodies targeting distinct epitopes on SARS-CoV-2 spike protein, thereby reducing viral entry and limiting the emergence of escape variants [18]. REGEN-COV was issued EUA by FDA on 21 November 2020, for the treatment of mild-to-moderate COVID-19 and high-risk patients. During subsequent phases of the pandemic, the EUA underwent multiple updates and finally was abolished on 24 January 2022 due to the surge of the Omicron variant and markedly reduced neutralization activity. Notwithstanding the later loss of authorization, REGEN-COV was widely used, with Regeneron reporting $6.19 billion attributable to REGEN-COV in full-year 2021 revenues.

Despite their clinical efficacy, accumulating evidence suggests that commonly used COVID-19 therapies may be associated with cardiovascular ADEs. For remdesivir, several studies have suggested a potential association between remdesivir use for COVID-19 treatment and subsequent cardiovascular events [19]. Some case reports or case series have documented sinus bradycardia, QT prolongation, T-wave abnormalities, QRS widening, and, in rare cases, complete atrioventricular (AV) block or cardiac arrest. An observational study by Grein et al. involving 61 hospitalized COVID-19 patients with oxygen saturation ≤94% reported that four patients experienced hypotension and atrial fibrillation after the use of remdesivir [20]. Similarly, Gupta et al. described cardiovascular events in two patients with coronavirus infection: both developed sinus bradycardia, and one exhibited prolonged corrected QT interval and T-wave abnormalities [21]. A single-center prospective observational study conducted over four months at Cotugno Hospital in Naples, Italy, examined 166 COVID-19 patients [22], of which 100 received remdesivir, while 66 served as controls. The incidence of bradycardia was significantly higher in the remdesivir group compared to controls (21% vs. 3%; *p* = 0.001). Additionally, Rafaniello et al. analyzed 1375 Individual Case Safety Reports (ICSRs) from the EudraVigilance database and found cardiac events in 8.4% of cases, some with fatal outcomes [23]. The cardiac risk of remdesivir was higher compared to other COVID-19 therapies, including hydroxychloroquine, lopinavir/ritonavir, tocilizumab, and azithromycin [23,24].

For Paxlovid, in real-world outcome analyses, although relatively rare and less severe [25,26], several studies have found that Paxlovid was associated with neurologic-related adverse reactions, dysgeusia, kidney dysfunction and diarrhea [27,28,29,30]. Specifically, the cardiovascular safety considerations are related to the pharmacology of ritonavir. With potent inhibition of CYP3A4, nirmatrelvir/ritonavir creates substantial potential for clinically important drug–drug interactions (DDIs) with cardiovascular medications, including antiarrhythmics, anticoagulants, and statins [31]. In addition, a care report found that a 71-year-old patient developed bradycardia, syncopal episodes, and sinus pause after taking Paxlovid, further highlighting the cardiovascular risk of Paxlovid [32].

Regarding REGEN-COV, pharmacovigilance analyses and case reports suggested possible cardiovascular ADEs in certain contexts. For example, a FAERS-based disproportionality study focusing on monoclonal antibodies found that casirivimab/imdevimab was associated with hypertension (reporting odds ratio (ROR) = 3.728, 95% confidence interval (CI) = 3.182–4.366) and ischemic heart disease (ROR = 1.986, 95% CI = 1.451–2.718) [33]. In addition, a care report has described that a 30-year-old patient had symptoms of myocarditis, including hypotension, chest pain, and shortness of breath, after receiving casirivimab/imdevimab for 15 min [34]. Together, these studies underscore the potential association between cardiovascular ADEs and REGEN-COV.

Despite these signals, the existing studies have important limitations that leave a major gap for comparative safety assessment. First, most studies only evaluate a single agent or a single therapeutic class (e.g., monoclonal antibody only), limiting the ability to compare cardiovascular ADEs across commonly used COVID-19 therapies within a unified framework. Second, the robustness of signals was limited by small sample size and single-center designs. Finally, the lack of molecular-level investigation restrains the understanding of mechanisms underlying the association between cardiovascular ADEs and COVID-19 therapies. Together, these limitations motivate a systematic study that directly compares cardiovascular ADEs across multiple major COVID-19 therapies based on large-scale real-world data.

In this study, both FAERS and TriNetX EHRs were leveraged, which provide extensive coverage and robust sample sizes. FAERS is a global pharmacovigilance database that collects real-world reports of ADEs, offering valuable insights into potential drug-ADE associations [35]. As of November 2025, FAERS contained nearly 32 million reports, with over one million new submissions annually. More than 30,000 reports related to COVID-19 emergency-authorized products have been added to the database. TriNetX is a global EHR network aggregating real-time, de-identified health records from diverse healthcare organizations. The U.S. Collaborative Network in TriNetX includes over 150 million patient records from 71 institutions, while the Global Collaborative Network contributes approximately 200 million records from 146 international partners. Compared to pharmacovigilance data, EHRs are less susceptible to reporting bias and serve as a complementary source for real-world evidence.

In summary, this study aims to compare the association between three widely used COVID-19 therapies, remdesivir, Paxlovid, and REGEN-COV, and cardiovascular ADEs and investigate the association patterns of remdesivir using FAERS and TriNetX data. Additionally, transcriptomic analyses were performed to explore biological changes potentially relevant to the cardiovascular ADEs observed in patients treated with remdesivir.

## 2. Results

### 2.1. Compare the Risks of Cardiovascular Events Observed in Patients Treated with COVID-19 Drugs

First, a comparative analysis across three COVID-19 therapies, remdesivir, nirmatrelvir/ritonavir (Paxlovid), and casirivimab/imdevimab (REGEN-COV), was performed using both FAERS and TriNetX EHR datasets. Using FAERS, the top 10 ADEs of three drugs were summarized separately, shown in Table 1. To focus on drug-specific effects, certain COVID-19-related events (e.g., death, COVID-19, respiratory failure, dyspnea, hypoxia, decreased oxygen saturation, COVID-19 pneumonia, pneumonia) and issues related to drug use (e.g., therapy discontinuation, off-label use, product preparation errors, and product use problems) were excluded.

In the FAERS descriptive summary, cardiovascular ADEs appeared among the top 10 reported events for remdesivir, with bradycardia ranking 3rd, hypotension ranking 7th, and cardiac arrest ranking 10th. In contrast, cardiovascular ADEs were less prominent in the top 10 lists for Paxlovid and REGEN-COV.

To further describe the relative prominence of selected cardiovascular ADEs within each drug-specific FAERS profile, reporting ranks across the three drugs were summarized (Table 2). Bradycardia ranks 3rd among the top ADEs for remdesivir, but much lower for Paxlovid (106th) and REGEN-COV (62nd). Similarly, cardiac arrest ranks 10th for remdesivir, compared with 375th for Paxlovid and 119th for REGEN-COV. The rank of hypotension for REGEN-COV (10th) is close to remdesivir (7th), whereas Paxlovid ranks considerably lower (92nd).

However, these rankings are descriptive and should not be interpreted as direct measures of comparative signal strength, particularly given the substantial variation in total report counts across drugs. Accordingly, disproportionality analyses were conducted to facilitate quantitative cross-drug comparisons. Disproportionality analysis presented in Table 3 revealed that the risk of bradycardia in patients treated with remdesivir was approximately 30 times higher than in those treated with Paxlovid and 6 times higher than in those treated with REGEN-COV. Similarly, the risk of cardiac arrest associated with remdesivir was about 50 times higher than with Paxlovid and 5 times higher than with REGEN-COV. Although REGEN-COV showed a higher risk of hypotension compared with remdesivir, Paxlovid consistently demonstrated a much lower risk.

**Figure 1:**
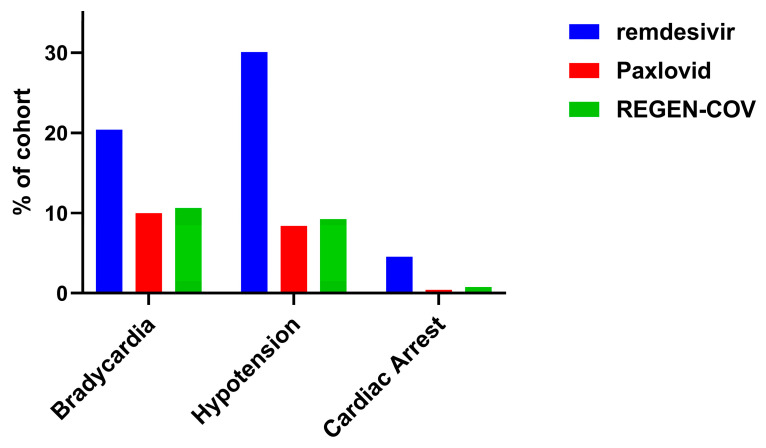
The comparison of three cardiovascular ADEs in patients treated with remdesivir, Paxlovid, and REGEN-COV using EHR data.

Analysis using TriNetX data revealed a similar trend. Among three COVID-19 therapies, patients received remdesivir exhibited the highest associations with cardiovascular ADEs. As shown in Figure 1, remdesivir was consistently associated with greater percentage of bradycardia (20.400% with remdesivir vs. 9.977% with Paxlovid vs. 10.645% with REGEN-COV), hypotension (30.084% with remdesivir vs. 8.394% with Paxlovid vs. 9.238% with REGEN-COV), and cardiac arrest (4.547% vs. 0.434% with Paxlovid vs. 0.771% with REGEN-COV). It should be noted that these prevalence comparisons were intended to provide a descriptive context with as these unmatched cohorts were not clinically equivalent control groups.

To provide the primary EHR comparison, relative risk estimation was conducted using propensity score-matched EHR data. Propensity score matching was applied using TriNetX built-in algorithm for cohort comparison to avoid biases caused by age, sex, and race. Table 4 shows that for all three cardiovascular ADEs, the RRs of remdesivir were consistently higher than Paxlovid and REGEN-COV. Specifically, for cardiac arrest, the risk for remdesivir was around 19 times higher than Paxlovid and around 12 times higher than REGEN-COV. Because the propensity score-matched TriNetX analyses were conducted as separate pairwise cohort comparisons, the eligible remdesivir denominator differed across unmatched, matched, and comparator-specific analyses.

Together, these findings suggest that, among the three COVID-19 therapies evaluated, remdesivir was associated with the higher observed risks of cardiovascular ADEs, especially bradycardia. However, these associations cannot be interpreted as the observed cardiovascular events being caused solely by remdesivir.

### 2.2. Validate the Associations Between Cardiovascular ADEs and Remdesivir Using FAERS and EHR

To validate the associations between cardiovascular ADEs and remdesivir, the 15 most frequently reported ADEs were assessed and summarized in Table 5. As of August 2025, 10,492 adverse event reports related to remdesivir were identified in the FAERS database. To reduce potential duplication, indirect reports originating from the literature were excluded, leaving 8143 cases for analysis. COVID-19-related events and drug use issues were excluded. In addition, in many FAERS records, patients were administered multiple drugs simultaneously. To determine whether the top ADEs were specifically associated with remdesivir or potentially caused by other medications, records of patients who received remdesivir only (single-drug cases) were also analyzed and compared with normal cases.

Table 5 shows that the five most common events of all cases group were increased alanine aminotransferase (*n* = 1028), increased aspartate aminotransferase (*n* = 702), bradycardia (*n* = 643), acute kidney injury (*n* = 554), and elevated liver function tests (*n* = 445). For better evaluation, the top 15 ADEs of both groups were broadly grouped into three major organ systems: hepatic, renal, and cardiovascular. Hepatic events included alanine aminotransferase increased, aspartate aminotransferase increased, liver function test increased, hepatic enzyme increased, and hypertransaminasaemia. Renal events comprised acute kidney injury, blood creatinine increased, renal impairment, and renal failure. Cardiovascular events included bradycardia, hypotension, and cardiac arrest.

**Figure 2:**
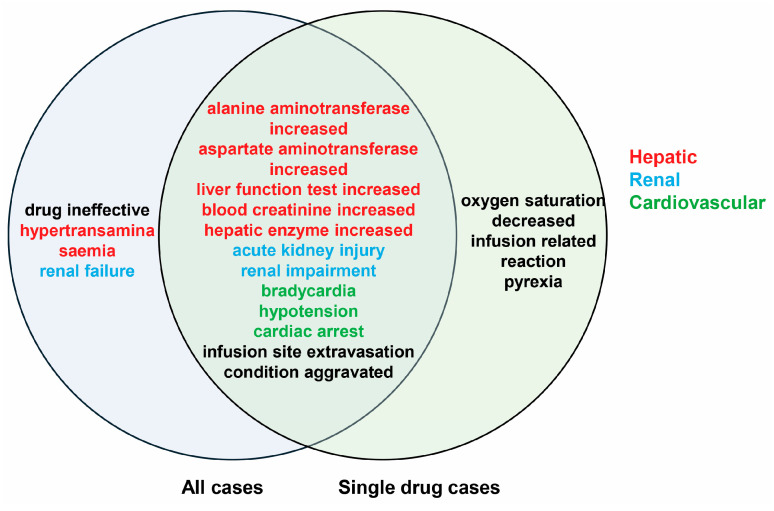
Top 15 ADEs reported with remdesivir in all cases and single-drug cases. Red: hepatic events; Blue: renal events; Green: cardiovascular events.

The ADE patterns of both groups were compared using Venn diagram, with different labels on ADEs of different organs. As is shown in Figure 2, the ADE patterns in the single-drug case group closely resemble those observed in the overall dataset. This descriptive similarity indicates that the major reported ADE categories were generally consistent across the two subsets. In addition, Table 5 shows that the top ADEs were highly similar across both groups, suggesting that the overall ADE pattern observed in remdesivir reports was not only driven by concomitant medications reported in multi-drug cases. However, the single-drug case analysis should be interpreted cautiously, because it remains descriptive and cannot exclude patient-level confounding or disease-related explanations.

To assess whether the cardiovascular ADEs identified in FAERS were associated with remdesivir treatment, EHR data from the TriNetX database were analyzed. The prevalence of three cardiovascular outcomes, bradycardia, hypotension, and cardiac arrest, were compared between patients who received remdesivir and those who did not. The prevalence comparison was intended to provide a descriptive context with a large sample size, with the limitation that the unmatched “without remdesivir” cohort cannot be interpreted as a clinically equivalent control group. Table 6 shows that the prevalence of bradycardia (18.559% vs. 2.473%), hypotension (26.836% vs. 2.267%), and cardiac arrest (4.357% vs. 0.399%) was markedly higher among remdesivir recipients.

The primary comparisons were conducted with propensity score matching between with remdesivir and without remdesivir cohorts. As is shown in Table 7, after matching, patients in the remdesivir cohort still had substantially higher risks for three cardiovascular ADEs. Compared with the crude prevalence shown in Table 6, the prevalences in both cohorts were lower after propensity score matching, likely because matching changed the underlying cohort composition. Although the absolute prevalences were reduced after matching, the relative risk estimates remained significant and consistently supported higher cardiovascular risks in the remdesivir cohort.

Together, these findings indicate a strong association between remdesivir use and increased risks of cardiovascular ADEs, especially bradycardia, hypotension, and cardiac arrest. However, these observational results do not establish that remdesivir itself is the only reason for these events, while potential confounders such as COVID-19 severity, cardiovascular risk, or concomitant medications remain possible.

### 2.3. Sex Differences in Cardiovascular Risks Among Remdesivir-Treated Patients

The potential sex differences in the risk of developing bradycardia, hypotension, and cardiac arrest were evaluated using FAERS and EHR data. Sex-stratified analyses included only reports with non-missing sex information. Therefore, denominators are smaller than the overall remdesivir FAERS cohort, and event counts do not sum to the full cohort totals. Table 8 shows the results of FAERS analysis, in which, bradycardia was reported in 261 of 2997 female patients and in 364 of 4737 male patients. The calculated OR (female as control group) was 0.873. with a 95% CI of 0.739–1.030, which includes 1, and a *p*-value of 0.108. The ORs (female as control group) for hypotension and cardiac arrest were greater than 1, but their corresponding *p*-values were not statistically significant.

Sex-stratified evaluation of cardiovascular ADEs’ risks was also conducted using EHR data and RRs were calculated upon each ADE. Table 9 shows that the prevalence of bradycardia was 21.463% in males and 19.592% in females, corresponding to a RR of 1.096 (95% CI: 1.079–1.112; *p* < 0.0001). For hypotension, the prevalence was 30.546% in males and 30.088% in females, with an RR of 1.015 (95% CI: 1.004–1.027; *p* = 0.0106). For cardiac arrest, the prevalence was 5.189% in males and 3.931% in females, yielding an RR of 1.320 (95% CI: 1.274–1.367; *p* < 0.0001). Among these three cardiovascular ADEs, the largest sex-related difference was observed for cardiac arrest, whereas the difference for hypotension was statistically significant but small in effect size.

Taken together, these results indicate that sex-related differences in remdesivir-associated cardiovascular ADEs were modest and not entirely consistent across data sources. Although male patients showed slightly higher observed risks in the EHR analysis, the FAERS analysis did not demonstrate significant sex-based differences. Therefore, the current evidence does not support a strong or definitive sex-specific pattern, and the subgroup results should be interpreted cautiously.

### 2.4. Age Differences in Cardiovascular Risks Among Remdesivir-Treated Patients

**Figure 3:**
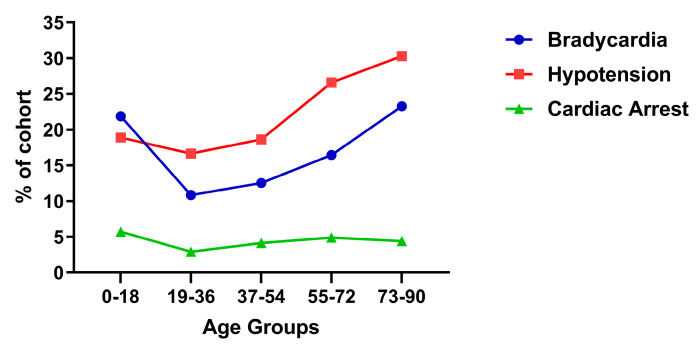
The prevalence of three cardiovascular ADEs in patients from five age groups treated with remdesivir using EHR.

To elucidate the age relationship with remdesivir-associated cardiovascular ADEs, EHR was applied for age-stratified prevalence comparison, while FAERS database does not contain enough reports. Remdesivir-treated patients were divided into five age groups (0–18, 19–36, 37–54, 55–72, 73–90 years). Table 10 and Figure 3 illustrate that bradycardia prevalence was highest among adolescents (21.867%), declined to about 10% in the 19–36 age group, and then progressively increased with advancing age, reaching a peak of 23.270% in the oldest cohort. Hypotension demonstrated an age-related gradient, rising steadily from 16.650% in younger adults to 30.290% in elderly. In contrast, the prevalence of cardiac arrest varied only modestly across age groups (2.907–5.711%), suggesting a comparatively weak age-related pattern.

To further evaluate age-related subgroup patterns, binomial logistic regression was performed with age group used as an ordered variable. Age group was coded as an ordered variable (1–5 corresponding to 0–18, 19–36, 37–54, 55–72, and 73–90 years). Table 11 summarizes the key regression parameters, including the regression coefficients (*β*_1_), standard error (SE), Z statistic (z), OR per age-group increment, 95% CI, and *p*-value for each cardiovascular ADE. OR presents the change in odds associated with a one-step increase in age group.

For bradycardia, the regression coefficient was positive (*β*_1_ = 0.246), and the OR per age-group step was 1.279 (95% CI: 1.263–1.294, *p* < 0.0001), indicating an overall age-associated increase in event frequency. However, this result should be interpreted carefully because the 0–18 years group for bradycardia showed a noticeable deviation from the general pattern observed across the adult age groups. For hypotension, a similarly positive association with age was observed with *β*_1_ as 0.240 and an OR of 1.271 (95% CI: 1.257–1.284, *p* < 0.0001). For cardiac arrest, the regression coefficient was much smaller (*β*_1_ = 0.0245), and the OR was 1.025 (95% CI: 1.004–1.047, *p* = 0.0214). Thus, although the age-associated effect reached statistical significance, the effect size was small, with an OR very close to 1, indicating only a modest age-related increase. Together, these results support age-associated variation across the three cardiovascular ADEs. The age-associated pattern was strongest for hypotension, remained substantial but non-uniform for bradycardia, and was comparatively weak for cardiac arrest.

### 2.5. Transcriptomic Studies of Remdesivir-Related Genes

An RNA-seq dataset including gene expression profiles of hESC-CMs treated with remdesivir was provided by Dr. Cao et al. from their previously published studies [36]. Our secondary data analysis identified 963 up-regulated and 623 down-regulated genes in remdesivir-treated human cardiomyocytes compared with untreated controls, using thresholds of FDR < 0.01 and fold change > 2.

Analysis of enriched pathways from 963 up-regulated genes identified several pathways related to cardiovascular biology, which provide supportive biological context for the cardiovascular safety signals observed in the FAERS and EHR analyses (Table 12). First, the cGMP–PKG signaling pathway was found to be significantly enriched, with a enrich fold of 3.12 and a *p*-value of 1.42 × 10^−3^, which may be relevant to vascular tone regulation and therefore may provide supportive context for the hypotension signal. Second, the dilated cardiomyopathy pathway was also significantly enriched, with a fold enrichment of 3.30 and a *p*-value of 1.18 × 10^−2^, suggesting transcriptomic changes related to cardiac structure and electrophysiology that may be relevant to arrhythmia-related outcomes. However, these pathway findings should be interpreted as hypothesis-generating rather than as a direct mechanistic explanation.

To provide additional biological context, enriched pathways of 623 down-regulated genes were identified (Table 13). In contrast, enriched pathways of down-regulated genes were predominantly enriched for metabolic and biosynthetic processes, including cholesterol metabolism, steroid hormone biosynthesis, amino acid biosynthesis and metabolism, metabolism of xenobiotics by cytochrome P450, glycolysis/gluconeogenesis, and carbon metabolism. As these pathways were mainly metabolism-related and did not map directly onto cardiovascular pathways, we did not interpret them as direct mechanistic explanations for cardiovascular ADEs identified. Instead, these transcriptomic findings were used to provide broader biological context and to inform future mechanistic and functional studies.

## 3. Discussion

In this study, we analyzed cardiovascular ADEs associated with remdesivir, Paxlovid, and REGEN-COV using two complementary data sources: FAERS pharmacovigilance reports and TriNetX EHRs. While FAERS provides valuable insights into spontaneously reported ADEs, it is subject to voluntary reporting bias and underreporting, which can limit the accuracy of prevalence estimates. In contrast, EHRs offer a substantially larger dataset and capture real-world clinical encounters, enabling more reliable prevalence estimation and reducing reporting bias. The propensity score-matched pairwise comparisons were considered the primary comparative analysis, whereas the unmatched prevalence comparison was included to provide descriptive background. The use of large-scale evidence across two real-world data sources greatly improves credibility of findings, compared to studies relying on either spontaneous reports or EHR data alone. The consistency of ADE patterns observed across both FAERS and TriNetX strengthens the validity of our findings and underscores the robustness of our conclusions. This alignment suggests that the identified safety signals are unlikely to be explained by a single data source and may reflect consistent real-world patterns. Importantly, although the cross-database consistency strengthens confidence in the observed association, it should not be interpreted as demonstrating specificity or causality for remdesivir itself.

Comparative analysis of FAERS data indicates that, compared with Paxlovid and REGEN-COV, remdesivir is the COVID-19 drug that is most frequently associated with cardiovascular ADEs, including bradycardia, hypotension, and cardiac arrest, a pattern further validated by TriNetX real-world evidence. This observation is complemented by the study of Rafaniello et al. [23]. Their disproportionality analyses based on ICSRs from the EudraVigilance database demonstrated a two-fold increased likelihood of reporting cardiac ADEs with remdesivir relative to hydroxychloroquine and azithromycin. This systemic analysis evaluates cardiovascular ADE patterns across multiple COVID-19 therapies with distinct pharmacology and therapeutic classes, an intravenous nucleotide prodrug antiviral (remdesivir), an oral protease inhibitor boosted by a potent CYP3A inhibitor (Paxlovid), and a neutralizing monoclonal antibody cocktail (REGEN-COV). The design provides an internal clinical comparison within the same disease context and helps explore whether observed cardiovascular events are a drug-specific safety signal or the broader cardiovascular manifestations of SARS-CoV-2 infection. In contrast, remdesivir-focused studies frequently evaluate a single agent and therefore cannot directly elucidate the magnitude or specificity of cardiovascular signals against other therapies [23,24,37].

The findings of remdesivir are consistent with the systematic review by Nabati et al. [19], which synthesized data from over ten studies. They identified bradycardia, hypotension, cardiac arrest, and QT interval prolongation as the most clinically significant cardiac complications linked to remdesivir therapy. Complementing these clinical observations, in vitro experiments by Choi et al. [38] revealed that remdesivir exerts marked cytotoxic effects on COVID-19-infected hESC-CMs, whereas chloroquine did not exhibit comparable cardiotoxicity in the same model. Importantly, extended exposure to remdesivir in cell-based assays substantially reduced hESC-CM viability, suggesting a potential risk of cumulative cardiotoxicity with prolonged treatment. These studies, together with our findings, highlight a potential association between remdesivir use and cardiovascular adverse events.

Remdesivir is a monophosphoramidate prodrug [39]. Its active metabolite, an adenosine nucleotide analog, is incorporated into viral RNA by the viral RNA-dependent RNA polymerase, where it causes delayed chain termination and inhibits viral RNA synthesis. This metabolite has a significant longer half-life than adenosine [19] and may contribute to bradycardia by activating A1 receptors in the AV node, leading to potassium ion efflux and reduced conduction [40]. In addition, this metabolite can stimulate nitric oxide production and subsequently increase cyclic guanosine monophosphate (cGMP) levels, which serve as a second messenger to promote vasodilation and hypotension. This agrees with our transcriptomic analysis that cGMP-PKG signaling pathway was significantly enriched in up-regulated genes by remdesivir in hESC-CMs. By contrast, Paxlovid acts as a selective SARS-CoV-2 main protease inhibitor designed with strong off-target selectivity, and the major cardiovascular concern is pharmacokinetic DDIs driven by ritonavir’s potent CYP3A inhibition [31,41]. Likewise, REGEN-COV is an extracellular neutralizing monoclonal antibody cocktail that binds nonoverlapping epitopes on the spike RBD to block viral entry, a modality that is not expected to directly mimic endogenous nucleoside signaling or engage cardiac receptors. These pharmacologic and transcriptomic observations may provide biological context for the observed association between remdesivir exposure and cardiovascular ADE signals, but they do not establish a drug-specific causal mechanism.

In addition, the safety profile of remdesivir may be influenced not only by the active drug itself but also by formulation-related excipients. In particular, remdesivir formulations contain sulfobutyl ether-β-cyclodextrin (SBECD), a solubilizing agent that may accumulate in patients with renal insufficiency and has been associated with renal and hepatic toxicity in previous pharmacologic studies [42]. Although this issue is outside the primary cardiovascular focus of the present analysis, it remains relevant to the broader clinical safety interpretation of remdesivir use.

Our pathway analysis upon remdesivir-treated hESC-CMs revealed significant enrichment of dilated cardiomyopathy and calcium signaling pathways among the up-regulated genes. Dilated cardiomyopathy, characterized by left ventricular enlargement and impaired systolic function, is a well-established risk factor for arrhythmia and sudden cardiac arrest [43]. Similarly, calcium signaling plays a central role in cardiac electrophysiology and contractility, and its dysregulation can lead to electrical instability, delayed afterdepolarizations, and life-threatening arrhythmias [44]. Given the established roles of these pathways in cardiac electrophysiology and contractility, these transcriptomic changes may provide supportive biological context for the observed associations with bradycardia, hypotension, and cardiac arrest in the FAERS and EHR analyses. In contrast, the enriched pathways of down-regulated genes were enriched mainly for metabolic processes. We therefore chose not to overinterpret these findings as direct mechanistic explanations for cardiovascular ADEs but present them as broader biological changes that may inform future targeted studies. In summary, our transcriptomic results provide supportive biological context for the observed associations between remdesivir and cardiac ADEs. However, these findings should not be interpreted as establishing a direct mechanistic link to cardiovascular outcomes.

Despite these findings, the present study has several limitations that warrant careful interpretation. First, due to the nature of observational studies, the findings can only suggest association between remdesivir and cardiovascular events and cannot establish a causal relationship.

Second, cross-therapy comparisons may be confounded by differences in clinical indication, efficacy, and underlying patient population across COVID-19 treatments. For example, remdesivir may have lower efficacy compared with other antiviral agents, or it may have been administered predominantly to patients with more severe COVID-19. Such patients typically exhibit greater systemic inflammation, multi-organ involvement, and higher baseline cardiovascular risk, all of which can increase the incidence of cardiovascular complications. Consequently, the increased prevalence of cardiac ADEs observed in the remdesivir group may partially reflect underlying disease severity rather than a drug-specific effect. This emphasizes the importance of considering illness severity when comparing safety profiles across COVID-19 therapies.

Third, an important limitation specific to our three-drug comparison is that remdesivir, Paxlovid, and REGEN-COV were deployed at different phases of the pandemic, when circulating variants, vaccination coverage, baseline disease severity, and clinical management strategies were changing. For example, real-world treatment patterns shifted substantially during 2022, with oral antivirals (including nirmatrelvir/ritonavir) replacing monoclonal antibodies as dominant outpatient therapies in several care settings [45]. In addition, the clinical utility of specific monoclonal antibodies was highly variant-dependent [46]. Specifically, REGEN-COV authorization was restricted by FDA when Omicron predominated because of markedly reduced activity against it. On the contrary, remdesivir was introduced earlier and used predominantly in hospitalized patients, with utilization evolving across waves [47]. Thus, differences in observed cardiovascular ADE patterns across three therapies could be attributed to time-varying patients and background risks rather than drug effects.

Fourth, intrinsic limitations of our two real-world data sources constrain the extent to which we can address the above biases. Neither FAERS nor TriNetX data contains key patient-level variables required to determine pandemic phase and clinical context, including confirmed viral lineage, vaccination status, standardized severity measures, physical condition, cardiovascular history, comorbidities, hospitalization status, ICU status, and concomitant medications. These missing factors limit our ability to precisely control cohort bias and avoid patient-, phase- and variant-related confounding factors. Consequently, our analyses should be interpreted carefully and future studies incorporating phase- and variant-stratified with well-established cohort matching designs are highly required.

Finally, mechanistic inference from the transcriptomic analysis is limited by the in vitro model used. Although the transcriptomic data derived from hESC-CMs provides valuable insight into cardiomyocyte-specific molecular responses, it cannot reflect the complex physiological environment of the human body, because of the lack of systemic factors such as neurohormonal regulation, vascular interactions, and immune responses that influence cardiovascular function in vivo. Consequently, certain critical pathways involved in cardiac remodeling, arrhythmogenesis and cardiac arrest may not be fully captured in this model. Therefore, the pathways and biological context suggested by our findings require further validation through in vivo studies and clinical investigations to confirm their relevance and to better understand the potential cardiovascular risks associated with remdesivir.

## 4. Materials and Methods

The public FAERS dashboard was utilized to search and download FDA FAERS records. These records from the FAERS dashboard contain the following information: drug information, drug adverse events, patient outcome for the reported adverse event, patient demographic information (such as patient sex, age, and body weight), source of the reported adverse event, reporting dates, and the indications for use.

Drug adverse events that were suspected to be related to a drug of interest were extracted from the FAERS dashboard by inputting the generic name of the drug (for instance, remdesivir) and known brand names (such as Veklury). The “suspect product names” or “suspect product active ingredients” of a record containing those generic or brand names of the drug (or salt of the drug) will be output from the FAERS dashboard.

Most of the FAERS records are directly from patients reported to FDA, a pharmaceutical company, or a health profession. There are also some indirect records extracted from publications. Those records were excluded in this study for two reasons. First, these reports are generally reported to FDA by multiple companies which would result in 2~10 duplicated copies for the same patients. Inclusion of these duplicated records would subsequently cause false positives in further calculation. Second, the reports from different companies may be inconsistent because different people have different understandings for the same publication or report.

EHRs were accessed and analyzed using real-world data network TriNetX (https://live.trinetx.com/). US Collaborative Network was selected as the dataset. Cohort remdesivir was defined by “RxNorm 2284718 Remdesivir”. Cohort Paxlovid was defined by “RxNorm 2587892 Nirmatrelvir” and “RxNorm 85762 Ritonavir”. Cohort REGEN-COV was defined as “HCPCS Q0243 Injection, casirivimab and imdevimab, 2400 mg” or “HCPCS Q0244 Injection, casirivimab and imdevimab, 1200 mg” or “HCPCS Q0240 Injection, casirivimab and imdevimab, 600 mg” or “HCPCS M0243 Intravenous infusion or subcutaneous injection, casirivimab and imdevimab includes infusion or injection, and post administration monitoring”. Patient numbers, demographic data, and clinical outcomes were all accessed through the TriNetX platform. The unmatched non-remdesivir cohort was used only for descriptive prevalence context and was not considered a clinically equivalent control for primary inference.

Age- and sex-stratified FAERS and EHR analyses were restricted to cases with available age and sex information. Therefore, the denominators are smaller than the full FAERS and EHR remdesivir cohort and subgroup counts do not sum to the overall totals.

Binomial logistic regression was conducted using age group as an ordered categorical predictor for cardiovascular ADEs’ prevalence trend analysis. Age was stratified into five groups (0–18, 19–36, 37–54, 55–72, and 73–90 years), which were coded sequentially from 1 to 5. Separate regression models were fitted for each ADE, with the presence or absence of each event as the binary outcome. The model was specified as

logit (*p*) = *β*_0_ + *β*_1_ × AgeGroup (1)

where *p* denotes the probability of the event within each age stratum. The exponentiated regression coefficient, *e^β^*^1^, was interpreted as the odds ratio (OR) per one-step increase in age group. For each model, ORs, 95% CI, and *p*-values were calculated to evaluate whether event frequency showed a significant age-associated trend across ordered age strata. Because age group was modeled as an ordered predictor, this analysis was intended to assess overall age-associated trend patterns rather than non-linear differences between individual age strata.

Disproportionality analyses were conducted to compare the risk of cardiovascular adverse events between the experimental group and the control group. The OR for these comparisons were then calculated using the standard formula:

OR = (a × d)/(b × c)(2)

where

a = number of events in the exposed group,

b = number of events in the control group,

c = number of non-events in the exposed group,

d = number of non-events in the control group.

A lower bound value greater than 1.0 for the 95% CI was indicative of a statistically significant, higher likelihood of reporting an adverse event in the experimental group than in the control group. *p*-values were calculated using chi-square test.

For EHR analysis, cardiovascular adverse events were evaluated using a cohort-based risk comparison framework. For each cardiovascular outcome, the cumulative incidence in the remdesivir cohort was compared with that in the propensity score matched-Paxlovid or REGEN-COV cohort. Relative risk (RR) was calculated as

RR = (a/(a + c))/(b/(b + d))(3)

where

a = number of patients with the event in the comparison cohort,

b = number of patients with the event in the remdesivir cohort,

c = number of patients without the event in the comparison cohort,

d = number of patients without the event in the remdesivir cohort.

A lower bound value greater than 1.0 for the 95% CI was indicated of a statistically significant, higher event risk in the comparison cohort relative to in the remdesivir cohort. *p*-value was calculated using chi-square test.

For propensity score matching, cohorts were matched using propensity scores on age at index, gender (Male, Female), and race (White, Black, or African American, and Asian) and compared separately. TriNetX applies 1:1 nearest-neighbor propensity score matching using the user-selected covariates. The platform performs matching automatically, and matched cohorts are balanced on these variables. Because the TriNetX analyses were conducted as separate pairwise cohort comparisons, the eligible remdesivir denominator differed across unmatched, matched, and comparator-specific analyses.

Transcriptomic data was provided by Dr. Cao et al. from their previously published studies [36]. In their experiments, h1 human embryonic stem cells (hESCs) were differentiated into cardiomyocytes and exposed to 10 μM remdesivir for 5 days with DMSO exposure as control. Three independent biological experiments were conducted for both remdesivir and control groups. Total RNA was extracted and amplified transcriptome libraries were sequenced on the Illumina Novaseq platform. Clean reads were then mapped against the human reference genome (GRCh38) using HISAT2 (v.2.1.0) software to generate read alignments for each sample. The quantification of gene expression was performed using the featureCounts v.1.6.0. Differentially expressed genes were identified using the limma package in R (version 4.5.2) with the thresholds as FDR < 0.01 and fold change > 2, and pathway enrichment analysis was performed upon up-regulated and down-regulated genes respectively using the ShinyGO web server.

## 5. Conclusions

This study provides convergent real-world evidence showing that remdesivir is the COVID-19 therapy that is most strongly and consistently associated with cardiovascular ADEs, when compared with Paxlovid and REGEN-COV, using two complementary data sources: FAERS reports and TriNetX EHRs. Furthermore, FAERS data indicated that the most frequently reported cardiovascular events linked to remdesivir were bradycardia, hypotension, and cardiac arrest. Importantly, the findings suggest that remdesivir-treated patients had higher observed cardiovascular event risks than COVID-19 patients treated with other medications. Sex-stratified using FAERS and EHR cannot support sex-dependent patterns for cardiovascular ADEs, while age-stratified analyses suggested age-associated variation in cardiovascular ADE frequency. Bradycardia showed a non-uniform pattern with higher prevalence in the youngest and oldest age groups, hypotension showed the clearest overall age-associated increase across adult age groups but with a noticeable deviation in the 0–18 years subgroup, and cardiac arrest showed only a weak age-associated effect. Transcriptomic analysis using RNA-seq data of hESC-CMs treated with remdesivir identified 963 up-regulated genes and 623 down-regulated genes. Pathway enrichment analysis upon up-regulated genes identified “cGMP-PKG signaling pathway”, “dilated cardiomyopathy”, and “calcium signaling pathway” as enriched pathways. In summary, our integrated analysis of pharmacovigilance, EHR, and transcriptomic data supports an association between remdesivir exposure and cardiovascular ADE signals while providing hypothesis-generating biological context.

## Figures and Tables

**Table 1:** The 10 most frequently reported ADEs by patients who took remdesivir, Paxlovid, and REGEN-COV.

	Remdesivir (*n* = 8143)		Paxlovid (*n* = 50,664)		REGEN-COV (*n* = 4172)	
No	Adverse Events	Frequency	Adverse Events	Frequency	Adverse Events	Frequency
1	alanine aminotransferase increased	1028	disease recurrence	19,817	infusion related reaction	874
2	aspartate aminotransferase increased	702	dysgeusia	7285	nausea	412
3	bradycardia *	643	diarrhea	3953	dizziness	359
4	acute kidney injury	554	nausea	2585	pyrexia	301
5	liver function test increased	445	headache	2029	chest discomfort	293
6	blood creatinine increased	354	cough	1947	chills	263
7	hypotension	240	fatigue	1854	flushing	238
8	hepatic enzyme increased	223	vomiting	1377	cough	232
9	renal impairment	209	nasal congestion	1325	hyperhidrosis	214
10	cardiac arrest	201	malaise	1273	hypotension	211

* Bradycardia included terms “bradycardia” and “sinus bradycardia”

**Table 2:** Comparison of the rank of reported cardiovascular ADEs among patients treated with remdesivir, Paxlovid, and REGEN-COV.

	Rank of Bradycardia	Rank of Hypotension	Rank of Cardiac Arrest
remdesivir	3	7	10
Paxlovid (without remdesivir)	106	92	375
REGEN-COV (without remdesivir)	62	10	119

**Table 3:** Disproportionality analysis of the frequencies of reported cardiovascular ADEs among patients treated with remdesivir, Paxlovid, and REGEN-COV using FAERS.

**Drugs**	**With Bradycardia**	**Without Bradycardia**	**OR** * **(95% CI) & *p*-Value**
remdesivir	643	7500	
Paxlovid (without remdesivir)	156	50,508	0.0360 (0.0302–0.0430), *p* < 0.0001
REGEN-COV (without remdesivir)	57	4115	0.162 (0.123–0.212), *p* < 0.0001
	**With Hypotension**	**Without Hypotension**	**OR (95% CI) & *p*-Value**
remdesivir	240	7903	
Paxlovid (without remdesivir)	182	50,482	0.119 (0.0978–0.144), *p* < 0.0001
REGEN-COV (without remdesivir)	211	3961	1.754 (1.452–2.119), *p* < 0.0001
	**With Cardiac Arrest**	**Without Cardiac Arrest**	**OR (95% CI) & *p*-Value**
remdesivir	201	7942	
Paxlovid (without remdesivir)	26	50,638	0.0203 (0.0135–0.0305), *p* < 0.0001
REGEN-COV (without remdesivir)	20	4152	0.190 (0.120–0.302), *p* < 0.0001

* Remdesivir as reference for calculating ORs.

**Table 4:** Relative risk estimation of reported cardiovascular ADEs among patients treated with remdesivir, Paxlovid, and REGEN-COV using propensity score-matched EHR.

**Drugs**	**With Bradycardia**	**Without Bradycardia**	**RR** * **(95% CI) & *p*-Value**
remdesivir	14,175	235,451	
Paxlovid	6471	243,155	0.457 (0.444–0.470), *p* < 0.0001
remdesivir	3181	66,236	
REGEN-COV	1173	68,244	0.369 (0.345–0.394), *p* < 0.0001
	**With Hypotension**	**Without Hypotension**	**RR (95% CI) & *p*-Value**
remdesivir	25,551	224,075	
Paxlovid	4990	244,636	0.195 (0.190–0.201), *p* < 0.0001
remdesivir	6214	63,203	
REGEN-COV (without remdesivir)	1128	68,289	0.182 (0.171–0.193), *p* < 0.0001
	**With Cardiac Arrest**	**Without Cardiac Arrest**	**RR (95% CI) & *p*-Value**
remdesivir	3053	246,573	
Paxlovid (without remdesivir)	165	249,461	0.054 (0.046–0.063), *p* < 0.0001
remdesivir	785	68,632	
REGEN-COV (without remdesivir)	65	69,352	0.083 (0.064–0.107), *p* < 0.0001

* Remdesivir as reference for calculating RRs.

**Table 5:** The 15 most frequently reported ADEs with remdesivir.

	All Cases (*n* = 8143)		Single-Drug Cases (*n* = 3862)	
No	Adverse Events	Frequency	Adverse Events	Frequency
1	alanine aminotransferase increased	1028	alanine aminotransferase increased	511
2	aspartate aminotransferase increased	702	aspartate aminotransferase increased	349
3	bradycardia *	643	bradycardia	252
4	acute kidney injury	554	liver function test increased	205
5	liver function test increased	445	acute kidney injury	167
6	blood creatinine increased	354	blood creatinine increased	145
7	hypotension	240	hepatic enzyme increased	129
8	hepatic enzyme increased	223	infusion site extravasation	116
9	renal impairment	209	hypotension	92
10	cardiac arrest	201	infusion related reaction	77
11	drug ineffective	173	condition aggravated	72
12	hypertransaminasaemia	161	renal impairment	68
13	condition aggravated	155	cardiac arrest	65
14	renal failure	150	pyrexia	60
15	infusion site extravasation	144	renal failure	59

* Bradycardia included terms “bradycardia” and “sinus bradycardia”.

**Table 6:** The prevalence of three cardiovascular ADEs in patients treated with or without remdesivir using EHR.

	With Remdesivir (*n* = 255,100)	Without Remdesivir (*n* = 127,272,860)
bradycardia	18.559% (47,345)	2.473% (3,147,612)
hypotension	26.836% (68,459)	2.267% (2,885,628)
cardiac arrest	4.357% (11,115)	0.399% (507,700)

**Table 7:** The relative risks of three cardiovascular ADEs in patients treated with or without remdesivir using propensity score-matched EHR.

	With Remdesivir (*n* = 197,890)	Without Remdesivir (*n* = 197,890)	RR (95% CI) & *p*-Value
bradycardia	3.461% (6849)	0.474% (938)	0.137 (0.128–0.147), *p* < 0.0001
hypotension	7.257% (14,360)	0.615% (1218)	0.0848 (0.0800–0.0899), *p* < 0.0001
cardiac arrest	1.082% (2142)	0.0359% (71)	0.0331 (0.0262–0.0420), *p* < 0.0001

**Table 8:** Disproportionality analysis of the risks of reported cardiovascular ADEs between female and male patients using FAERS.

**Group**	**With Bradycardia**	**Without Bradycardia**	**OR** * **(95% CI) & *p*-Value**
Female	261	2736	
Male	364	4373	0.873 (0.739–1.030), *p* = 0.108
	**With Hypotension**	**Without Hypotension**	**OR (95% CI) & *p*-Value**
Female	86	2825	
Male	149	4439	1.102 (0.842–1.444), *p* = 0.478
	**With Cardiac Arrest**	**Without Cardiac Arrest**	**OR (95% CI) & *p*-Value**
Female	72	2853	
Male	128	4481	1.132 (0.845–1.517), *p* = 0.407

* Female as reference for calculating ORs.

**Table 9:** Relative risk analysis of the risks of reported cardiovascular ADEs between female and male patients using FAERS.

**Group**	**With Bradycardia**	**Without Bradycardia**	**Prevalence**	**RR** * **(95% CI) & *p*-Value**
Female	24,918	102,268	19.592%	
Male	29,461	107,803	21.463%	1.096 (1.079–1.112), *p* < 0.0001
	**With Hypotension**	**Without Hypotension**		**RR (95% CI) & *p*-Value**
Female	38,268	88,918	30.088%	
Male	41,928	95,336	30.546%	1.015 (1.004–1.027), *p* = 0.0106
	**With Cardiac Arrest**	**Without Cardiac Arrest**		**RR (95% CI) & *p*-Value**
Female	5000	122,186	3.931%	
Male	7122	130,142	5.189%	1.320 (1.274–1.367), *p* < 0.0001

* Female as reference for calculating RRs.

**Table 10:** Prevalence of reported cardiovascular ADEs between different age groups under remdesivir administration.

Group	With Bradycardia	Prevalence	With Hypotension	Prevalence	With Cardiac Arrest	Prevalence	Case Number
0–18	1164	21.867%	1005	18.880%	304	5.711%	5323
19–36	1396	10.821%	2148	16.650%	375	2.907%	12,901
37–54	2974	12.537%	4417	18.620%	976	4.114%	23,722
55–72	12,576	16.438%	20,350	26.599%	3733	4.879%	76,507
73–90	21,068	23.270%	27,424	30.290%	3979	4.395%	90,537

**Table 11:** Binomial logistic regression analysis of age-associated trends in cardiovascular adverse events across ordered age groups.

	*β*_1_	SE	z	OR Per Age-Group Step (95% CI) & *p*-Value
bradycardia	0.246	0.00623	39.430	1.279 (1.263–1.294), *p* < 0.0001
hypotension	0.240	0.00540	44.410	1.271 (1.257–1.284), *p* < 0.0001
cardiac arrest	0.0245	0.0107	2.300	1.025 (1.004–1.047), *p* = 0.0214

**Table 12:** Enrichment pathway analysis of 963 up-regulated genes under remdesivir treatment.

Pathway	No. of Genes	Fold Enrichment	Enrichment FDR	Genes
Insulin secretion	14	4.69	1.40 × 10^−4^	*ADCY1 ADCY6 ADCY7 ADCY9 KCNMB4 PCLO KCNJ11 ATP1A1 ATP1A2 ATP1B1 TRPM4 RAB3A RYR2 CACNA1D*
Lysine degradation	10	4.57	2.37 × 10^−3^	*MECOM SETD1B ASH1L KMT2E KMT2C PRDM16 NSD1 KMT2D SETD7 DOT1L*
Circadian entrainment	15	4.46	1.40 × 10^−4^	*ADCY1 ADCY6 ADCY7 ADCY9 GNG7 GRIN2A GUCY1A2 PER1 PRKG1 RYR2 CACNA1D PER3 CACNA1I CACNA1H CACNA1G*
Cortisol synthesis and secretion	9	3.99	1.02 × 10^−2^	*ADCY1 ADCY6 ADCY7 ADCY9 NCEH1 CACNA1D CACNA1I CACNA1H CACNA1G*
Pancreatic secretion	13	3.67	2.37 × 10^−3^	*CEL ADCY1 ADCY6 ADCY7 ADCY9 ATP1A1 ATP1A2 ATP1B1 ATP2A2 ATP2B4 PLA2G5 RYR2 SLC4A4*
Cardiac muscle contraction	11	3.64	6.63 × 10^−3^	*COX6A2 HRC MYH6 ATP1A1 ATP1A2 ATP1B1 ATP2A2 CACNG6 RYR2 CACNA1D SLC9A7*
Aldosterone synthesis and secretion	12	3.53	5.31 × 10^−3^	*ADCY1 ADCY6 ADCY7 ADCY9 ATP1A1 ATP1A2 ATP1B1 ATP2B4 CACNA1D CACNA1I CACNA1H CACNA1G*
Dilated cardiomyopathy	11	3.30	1.18 × 10^−2^	*ADCY1 ADCY6 ADCY7 ADCY9 LAMA2 MYH6 ATP2A2 CACNG6 RYR2 TTN CACNA1D*
Gap junction	10	3.27	1.93 × 10^−2^	*TUBB4A ADCY1 ADCY6 ADCY7 ADCY9 EGF GUCY1A2 PDGFB PRKG1 SOS1*
cGMP-PKG signaling pathway	18	3.12	1.42 × 10^−3^	*ADCY1 ADCY6 ADCY7 ADCY9 KCNMB4 GUCY1A2 MEF2A MYH6 ATP1A1 ATP1A2 NFATC3 ATP1B1 NOS3 ATP2A2 ATP2B4 PRKG1 CACNA1D PDE5A*
Vascular smooth muscle contraction	14	3.01	6.84 × 10^−3^	*ADCY1 ADCY6 ADCY7 ADCY9 ADM EDN2 ARHGEF12 KCNMB4 GUCY1A2 PPP1R12B PLA2G5 PRKCQ PRKG1 CACNA1D*
Calcium signaling pathway	25	3.00	1.40 × 10^−4^	*ADCY1 CHRM2 ADCY7 PLCD3 ADCY9 EGF ERBB4 FGF7 GRIN2A HRC HTR4 NOS3 ATP2A2 NTRK2 ATP2B4 PDGFB PHKA1 TPCN1 CYSLTR2 RYR2 TACR2 CACNA1D CACNA1I CACNA1H CACNA1G*

**Table 13:** Enrichment pathway analysis of 623 down-regulated genes under remdesivir treatment.

Pathway	No. of genes	Fold Enrichment	Enrichment FDR	Genes
Complement and coagulation cascades	37	17.648	1.78 × 10^−34^	*PROCR CLU CPB2 F2 F2RL2 F5 F7 F11 F12 F13B FGA FGB FGG SERPIND1 CFHR1 CFI KNG1 MBL2 SERPINC1 SERPINA5 SERPINA1 PLG SERPINF2 PROC CFB SERPING1 C1S C2 C3 C4BPA C4BPB C5 C6 C8A C8B VTN VWF*
Maturity onset diabetes of the young	8	12.621	3.62 × 10^−6^	*RFX6 NR5A2 HHEX FOXA2 FOXA3 HNF4A PKLR SLC2A2*
Cholesterol metabolism	15	12.065	7.22 × 10^−11^	*PCSK9 ANGPTL3 APOA1 APOA2 APOA4 APOB APOC1 APOC3 APOE APOH LIPC ANGPTL8 ABCG8 SOAT2 LIPG*
Steroid hormone biosynthesis	15	9.767	1.27 × 10^−9^	*COMT CYP3A7 CYP3A5 CYP19A1 AKR1C1 AKR1C2 HSD17B2 HSD17B11 AKR1D1 SULT1E1 UGT2B4 UGT2B7 UGT2B10 UGT2A3 AKR1C3*
Drug metabolism-cytochrome P450	16	9.376	6.66 × 10^−10^	*ADH1A ADH4 ADH6 CYP2C19 CYP2C9 CYP3A5 FMO1 GSTA1 GSTA2 AOX1 MAOB MGST2 UGT2B4 UGT2B7 UGT2B10 UGT2A3*
Arginine biosynthesis	5	8.917	2.99 × 10^−3^	*GLUD1 ARG1 ASL ASS1 OTC*
Fat digestion and absorption	9	8.585	1.70 × 10^−5^	*FABP1 NPC1L1 APOA1 APOA4 APOB ACAT2 MTTP ABCG8 PLA2G12B*
Tyrosine metabolism	7	7.976	4.00 × 10^−4^	*ADH4 ADH6 COMT DDC HGD AOX1 MAOB*
Retinol metabolism	13	7.842	3.53 × 10^−7^	*ADH1A ADH4 ADH6 CYP3A7 CYP2C9 CYP3A5 ALDH1A1 AOX1 UGT2B4 UGT2B7 UGT2B10 UGT2A3 ALDH1A2*
Chemical carcinogenesis-DNA adducts	13	7.729	3.73 × 10^−7^	*CYP3A7 CYP2C19 CYP2C9 CYP3A5 AKR1C2 GSTA1 GSTA2 MGST2 SULT2A1 UGT2B4 UGT2B7 UGT2B10 UGT2A3*
Metabolism of xenobiotics by cytochrome P450	14	7.556	1.83 × 10^−7^	*ADH1A ADH4 ADH6 CYP2C9 CYP3A5 AKR1C1 GSTA1 GSTA2 MGST2 SULT2A1 UGT2B4 UGT2B7 UGT2B10 UGT2A3*
PPAR signaling pathway	12	6.477	7.68 × 10^−6^	*SLC27A2 APOA5 DBI FABP1 HMGCS2 APOA1 APOA2 APOC3 AQP7 ACSL5 PPARG ACOX2*
Staphylococcus aureus infection	13	5.673	1.11 × 10^−5^	*FGG KRT23 HLA-DRB1 HLA-DRB5 CFI ITGAL MBL2 PLG CFB C1S C2 C3 C5*
Bile secretion	12	5.469	3.65 × 10^−5^	*SLCO1B1 SLC22A7 ABCC2 ABCG8 SLC5A1 SULT2A1 UGT2B4 UGT2B7 UGT2B10 UGT2A3 NR0B2 ABCC3*
Biosynthesis of amino acids	9	4.922	1.45 × 10^−3^	*ALDOB ARG1 MAT1A ASL ASS1 OTC PAH PC PKLR*
Glycolysis/Gluconeogenesis	8	4.898	3.14 × 10^−3^	*ADH1A ADH4 ADH6 FBP1 ALDOB G6PC1 PKLR HKDC1*
Cell adhesion molecules	16	4.128	3.65 × 10^−5^	*CDH5 JAML CLDN4 CLDN3 CLDN7 HLA-DRB1 HLA-DRB5 ITGAL PECAM1 SDC1 CLDN5 CLDN1 ESAM CD4 NRXN3 CD34*
Carbon metabolism	11	3.924	1.89 × 10^−3^	*GLYCTK AGXT FBP1 ALDOB GLDC GLUD1 ACAT2 PC PKLR HKDC1 RGN*
Coronavirus disease-COVID-19	22	3.857	2.43 × 10^−6^	*AGTR1 RPL22L1 F2 F13B FGA FGB FGG MBL2 OAS1 RPL34 RPS4Y1 CFB CCL2 C1S C2 C3 C5 C6 C8A C8B VWF CASP1*

## Data Availability

The original contributions presented in this study are included in the article. Further inquiries can be directed to the corresponding author.

## Abbreviations

The following abbreviations are used in this manuscript:

ADE	Adverse Drug Event
FDA	Food and Drug Administration
FAERS	FDA Adverse Event Reporting System
EHR	Electronic Health Record
hESC-CM	Human Embryonic Stem Cell-Derived Cardiomyocytes
SARS-CoV-2	Severe Acute Respiratory Coronavirus-2
RdRp	RNA-Dependent RNA Polymerase
EUA	Emergency Use Authorization
M^pro^	Main Protease
AV	Atrioventricular
ICSR	Individual Case Safety Report
DDI	Drug–Drug Interaction
RR	Relative Risk
OR	Odds Ratio
SE	Standard Error
ROR	Reporting Odds Ratio
CI	Confidence Interval
cGMP	Cyclic Guanosine Monophosphate
mAb	Monoclonal Antibody
SBECD	Sulfobutyl Ether-β-Cyclodextrin
